# Comparison of MRI radiomics-based machine learning survival models in predicting prognosis of glioblastoma multiforme

**DOI:** 10.3389/fmed.2023.1271687

**Published:** 2023-11-30

**Authors:** Di Zhang, Jixin Luan, Bing Liu, Aocai Yang, Kuan Lv, Pianpian Hu, Xiaowei Han, Hongwei Yu, Amir Shmuel, Guolin Ma, Chuanchen Zhang

**Affiliations:** ^1^Department of Radiology, Liaocheng People’s Hospital, Shandong First Medical University & Shandong Academy of Medical Sciences, Liaocheng, Shandong, China; ^2^China-Japan Friendship Hospital (Institute of Clinical Medical Sciences), Chinese Academy of Medical Sciences & Peking Union Medical College, Beijing, China; ^3^Department of Radiology, China-Japan Friendship Hospital, Beijing, China; ^4^Peking University China-Japan Friendship School of Clinical Medicine, Beijing, China; ^5^Department of Radiology, The Affiliated Drum Tower Hospital of Nanjing University Medical School, Nanjing, China; ^6^McConnell Brain Imaging Centre, Montreal Neurological Institute, McGill University, Montreal, QC, Canada; ^7^Department of Neurology and Neurosurgery, McGill University, Montreal, QC, Canada

**Keywords:** glioblastoma multiforme, radiomics, machine learning, survival models, prognosis

## Abstract

**Objective:**

To compare the performance of radiomics-based machine learning survival models in predicting the prognosis of glioblastoma multiforme (GBM) patients.

**Methods:**

131 GBM patients were included in our study. The traditional Cox proportional-hazards (CoxPH) model and four machine learning models (SurvivalTree, Random survival forest (RSF), DeepSurv, DeepHit) were constructed, and the performance of the five models was evaluated using the C-index.

**Results:**

After the screening, 1792 radiomics features were obtained. Seven radiomics features with the strongest relationship with prognosis were obtained following the application of the least absolute shrinkage and selection operator (LASSO) regression. The CoxPH model demonstrated that age (HR = 1.576, *p* = 0.037), Karnofsky performance status (KPS) score (HR = 1.890, *p* = 0.006), radiomics risk score (HR = 3.497, *p* = 0.001), and radiomics risk level (HR = 1.572, *p* = 0.043) were associated with poorer prognosis. The DeepSurv model performed the best among the five models, obtaining C-index of 0.882 and 0.732 for the training and test set, respectively. The performances of the other four models were lower: CoxPH (0.663 training set / 0.635 test set), SurvivalTree (0.702/0.655), RSF (0.735/0.667), DeepHit (0.608/0.560).

**Conclusion:**

This study confirmed the superior performance of deep learning algorithms based on radiomics relative to the traditional method in predicting the overall survival of GBM patients; specifically, the DeepSurv model showed the best predictive ability.

## 1 Introduction

Glioblastoma multiforme (GBM) is the most common and least prognostic primary tumour of the central nervous system, with a 5-year survival rate of 6–22% based on a combination of age at diagnosis and other risk factors (1). Prognostic models that include only the patient’s age, ethnicity, whether or not they receive radiotherapy, and risk factors such as the size, location and histopathological composition of the tumour often fail to predict overall survival (OS) accurately (2, 3). Therefore, identifying risk factors for GBM prognosis and developing appropriate predictive models are essential for the individualized and precise treatment of GBM patients.

Radiomics, which transforms digital medical images into mineable high-dimensional features and builds statistical models to analyze the features, has been widely used in tumour diagnosis, prognosis prediction, and treatment selection (4). Studies have shown that GBM radiomics information is closely related to patient prognosis and recurrence (5, 6). Zhang et al. (7) developed and validated a radiomics nomogram model to determine GBM survival probabilities in a non-invasive manner, achieving superior accuracy in both the training and test set. Survival analysis (also known as time-effect analysis) methods have been widely used in medical research, such as clinical efficacy trials and disease prognosis analysis. The Cox proportional-hazards model (Cox-PH) is the most well-known method used to determine the association between clinical predictor variables and the risk of mortality events. The CoxPH model is based on the assumption of a linear combination of event risk and variables; however, it is likely to be too simplistic to fit the actual disease progression.

Machine learning is a branch of artificial intelligence that has a wide range of applications in diagnosing and prognostic assessing GBM (5, 8). Compared to CoxPH models, machine learning can identify clinically significant risks with some marginal variables that can significantly improve the model’s performance (9). Deep learning (DL) is a frontier area of machine learning algorithms. Deep learning-based features are mainly extracted through convolutional neural networks (CNN), and feature learning algorithms are derived from the data itself and are more targeted to specific studies (10), and are widely used in imaging diagnosis, disease staging and prognosis, which can effectively improve outcome prediction (11–13). The Deepsurv model is a deep learning technique applied to a non-linear cox proportional risk network (14). Studies have shown that the DeepSurv model can obtain patient risk factors from multiple parameters and has achieved good predictive performance in assessing different patients, such as lung cancer and nasopharyngeal carcinoma (15, 16). Previous deep-learning algorithms that have been applied to assess the prognosis of GBM patients used traditional clinical prognostic risk factors and did not incorporate radiomics features (17). To our knowledge, no study has been conducted on the prognosis of GBM patients using radiomics combined with machine learning. Therefore, this study aimed to construct: (1) a traditional CoxPH model, (2) a tree-based SurvivalTree model, (3) an RSF model based on ensemble learning, (4) a DeepSurv, and (5) a DeepHit model based on deep learning for predicting the overall survival of GBM patients based on GBM radiomics and clinical data. Following the construction of these five models, we compared their performance.

## 2 Materials and methods

### 2.1 Clinical case data

**Figure 1:**
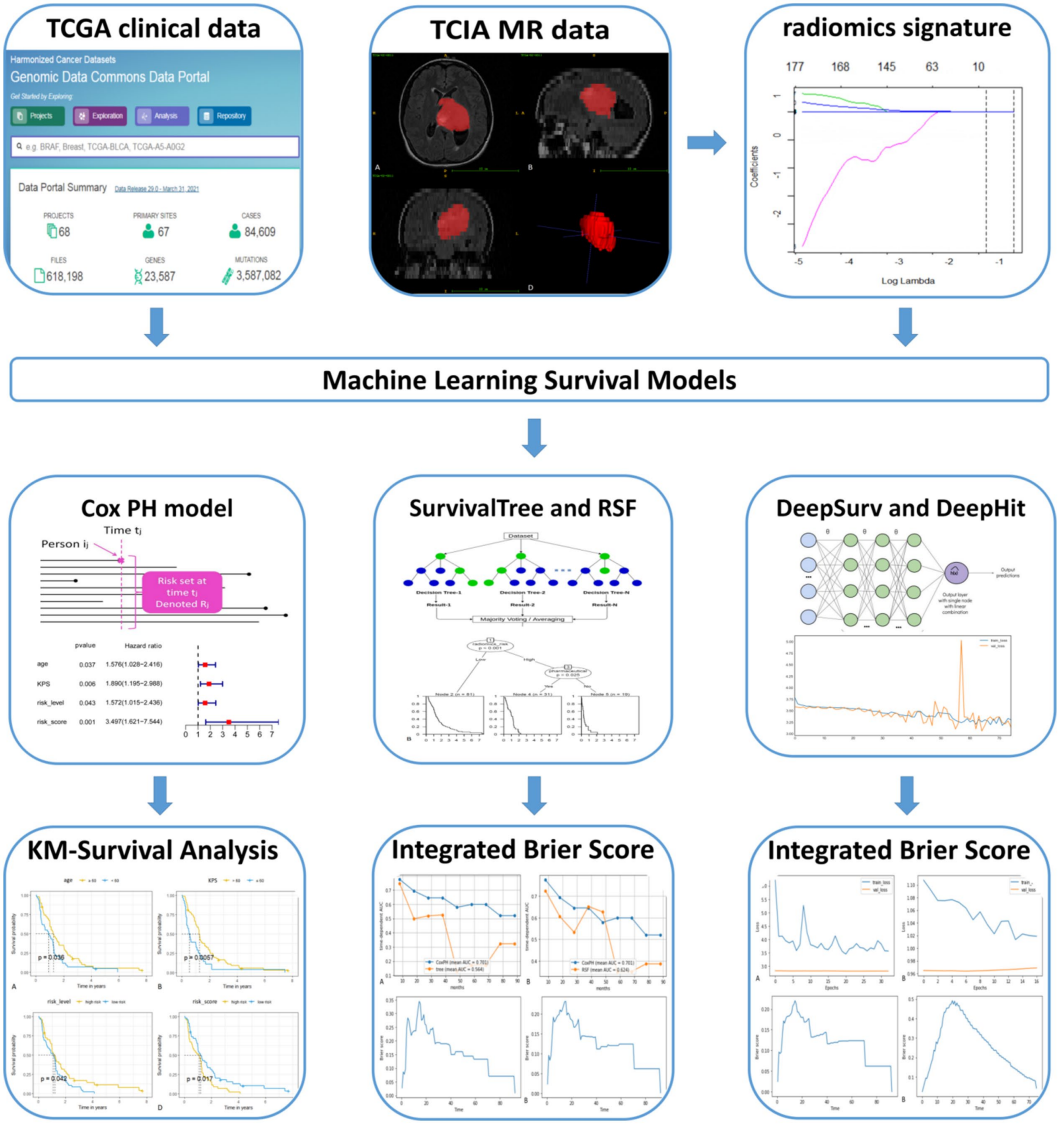
Flow chart of the study.

According to the proposed inclusion criteria, (1) clinical information of The Cancer Genome Atlas (TCGA) for GBM was downloaded from the TCGA database^1^ and (2) Magnetic Resonance Imaging (MRI) data were obtained from the Cancer Imaging Archive (TCIA),^2^ and a total of 262 patients were enrolled. Then, 131 patients were excluded due to (1) the lack of fluid-attenuated inversion recovery (FLAIR) sequences from TCIA (*n* = 114) and (2) MRI sequences acquired with severe motion or artefacts that may have induced bias in the subsequent analysis (*n* = 17). A total of 131 patients with GBM were subsequently retrospectively enrolled in our study. In this retrospective study, the requirement for informed consent was waived, as the relevant patient data in the TCGA were publicly available. We followed the relevant policies of the TCGA and TCIA in the acquisition and use of data. The flow chart for this study is shown in Figure 1.

### 2.2 Image acquisition and segmentation

**Figure 2:**
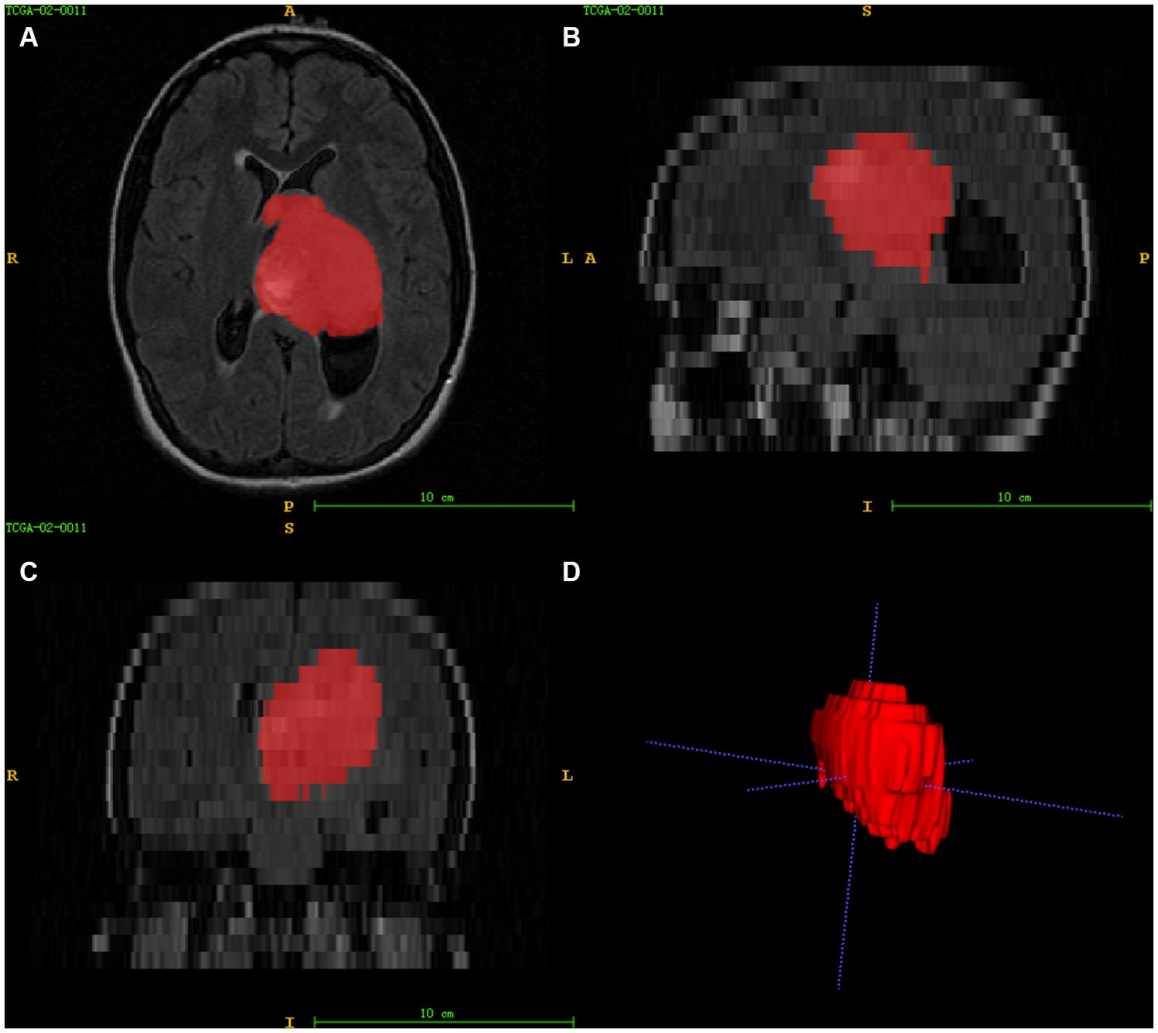
Image segmentation **(A–C)** represent the axial, sagittal, and coronal views of the images, respectively, and **(D)** shows the 3D reconstruction results of the ROI.

Using ITK-SNAP^3^ software to segment the FLAIR images of patients in 3D, the segmentation process is shown in Figure 2. The FLAIR scan parameters were as follows: thickness = 4 ~ 5.5 mm, TR/TE = 9,000 ~ 12,500/140 ~ 157 ms, slice gap = 4 ~ 6.5 mm, flip angle = 80 ~ 90°. The area of interest covered the entire tumour and edema region, and all feature extraction methods were implemented using the Cancer Imaging Phenomics Toolkit (CaPTk www.cbica.upenn.edu/captk). To confirm the reproducibility of the features, 30 patients were randomly selected, two people performed the Region Of Interest (ROI) segmentation, and the intraclass correlation coefficient (ICC) of the two ROIs was calculated (18). A threshold of ICC > 0.8 was set for considering a good agreement between the two neuro-radiologists. Features that achieved ICC higher than this thereshold were considered as showing reproducibility. The calculated features all contain first-order statistical features and statistical-based texture features, such as grey-level co-occurrence matrices (GLCM), grey-level dependence matrix (GLDM), neighbourhood grey-tone difference matrices (NGTDM), grey-level run-length matrices (GLRLM), and grey-level size zone matrices (GLSZ), grey-level size zone matrices (GLSZM) (19, 20).

### 2.3 Establishing radiomics signature and data cleaning

The least absolute shrinkage and selection operator (LASSO) method was used to select key features from the dataset significantly associated with prognosis. The selected features were linearly combined according to their respective coefficient weights to construct a radiomics signature, calculate the risk score for each patient, and determine the risk level. Subsequently, all collected data were classified as numerical or subtypes according to the input features. The missing data imputation was performed using the k-nearest neighbor (KNN) algorithm (Supplementary Table S1).

### 2.4 Feature engineering

According to Subtype, one-hot coding was performed to convert different categories of risk factors into categorical variables. This resulted in two new features called Subtype_Mesenchymal and Subtype_Proneural.

### 2.5 Construction of the model

#### 2.5.1 CoxPH model

For the CoxPH model, proportional risk assumptions were made using the CoxPHFitter function. Filter-based feature selection was performed using Cox regression to select features significantly associated with prognosis in GBM patients. All comparisons were performed at the 95% confidence level, with *p* < 0.05 indicating statistical significance.

#### 2.5.2 SurvivalTree model

SurvivalTree is based on classification and regression trees (CART) (21). The model is based on the tree structure, and the tree building mainly includes tree generation and pruning. Simple dichotomous classification problems can better perform the prognostic grouping of the method.

#### 2.5.3 RSF model

Random Survival Forest is a combination of random forest (RF) and survival analysis methods. The model calculates a cumulative risk function for each tree by selecting a subset of variables at each node and splitting the node tree based on survival time and event state, and finally calculates the mean of the integrated cumulative risk function to predict the error (22).

#### 2.5.4 DeepSurv model

DeepSurv is a feed-forward deep neural network for CoxPH models to model a nonlinear representation of the risk of clinical events based on input features. The model architecture includes network inputs from patient data, fully connected and hidden layers, and an output layer with linearly activated individual nodes for estimating the logarithmic risk function in the CoxPH model (14). DeepSurv can make predictions without specifying interaction terms, and in addition, the model’s hyperparameters can vary depending on the model’s performance.

#### 2.5.5 DeepHit model

The DeepHit model was initially designed to analyze the competing risks of multiple events (23). In the present study, we considered only one event: patient survival. Therefore, we can use a simplified DeepHit model to analyze our data. We can obtain an estimated probability value with the softmax layer of the model.

### 2.6 Model training and evaluation

After data preprocessing, the data was divided into 70% training data and 30% test data. The hyperparameters of the models were selected via random search. The performance of the models was compared using Harrell’s concordance index (C-index) and brier scores. C-index was used to estimate the proportion of random individuals with the same survival time ranking as their accurate survival time, with a C-index value of 1 indicating perfect discrimination and when 0.5 indicating random prediction. The brier score represents the mean squared difference between the observed patient status and the predicted probability of survival, with scores ranged from 0 (worst) to 1 (best). The overall estimate of the brier score for all available times is called the Integrated Brier Score (IBS). In practice, models with IBS below 0.25 are considered valuable. In addition, for the SurvivalTree and RSF models, we also used the receiver operating characteristic curves (ROC) over time and calculated the area under the curve (AUC) values to evaluate the model performance.

### 2.7 Statistical analysis

Statistical analysis was performed using R 3.6.0 and the model construction was performed using Python 3.7. The R packages used are as follows: glmnet package for LASSO logistic regression, gplots and pheatmap packages for heat map analysis. The Python packages were used are as follows: CoxPH analysis using the lifelines package, SurvivalTree and RSF using the scikit-survival package, feature importance ranking using the permutation_importance function; DeepSurv and DeepHit using the Pytorch-based pycox package. The comparison of patients between training and test set was performed for continuous variables with a t-test or Mann–Whitney test. The chi-square test was performed for subtype variables. All statistics were two-tailed, and *p*-values less than 0.05 were considered statistically significant.

## 3 Results

### 3.1 Clinical characteristics of patients

The clinical characteristics of the patients in the training and test set are shown in Table 1. There were no statistically significant differences in patient age, sex, race, radiation, pharmaceutical, survival status or survival month between the training and test set (*p* = 0.071–1.000).

**Table 1:** Demographics of patients enrolled in the training set and test set.

Variables		Total (*n* = 131)	Training set (*n* = 91)	Test set (*n* = 40)	*p*
Age					0.220
	≤60	73 (56%)	47 (52%)	26 (65%)	
	>60	58 (44%)	44 (48%)	14 (35%)	
Sex					0.979
	female	44 (34%)	30 (33%)	14 (35%)	
	male	87 (66%)	61 (67%)	26 (65%)	
Race					0.462
	white	20 (15%)	12 (13%)	8 (20%)	
	others	111 (85%)	79 (87%)	32 (80%)	
KPS					0.645
	≤60	93 (71%)	63 (69%)	30 (75%)	
	>60	38 (29%)	28 (31%)	10 (25%)	
Subtype					0.742
	Classical	36 (27%)	24 (26%)	12 (30%)	
	Proneural	49 (37%)	36 (40%)	13 (32%)	
	Mesenchymal	46 (35%)	31 (34%)	15 (38%)	
CIMP_status					0.773
	G-CIMP	116 (89%)	81 (89%)	35 (88%)	
	Non G-CIMP	15 (11%)	10 (11%)	5 (12%)	
Radiation					1.000
	no	102 (78%)	71 (78%)	31 (78%)	
	yes	29 (22%)	20 (22%)	9 (22%)	
Pharmaceutical					0.454
	no	101 (77%)	68 (75%)	33 (82%)	
	yes	30 (23%)	23 (25%)	7 (18%)	
Survival status					0.071
	alive	16 (12%)	8 (9%)	8 (20%)	
	dead	115 (88%)	83 (91%)	32 (80%)	
Survival months^#^		12.27 (5.5, 19.9)	13.13 (5, 22.09)	11.71 (6.88, 17.62)	0.581

^#^Continuous variables; median (range).

### 3.2 Radiomics feature extraction and construction of radiomics signature

In this study, 1792 radiomics features were obtained based on T2-FLAIR images from the TICA database, using CaPTk software. The 1792 features were brought into the LASSO cox regression model to screen the optimal radiomics features. We screened the optimal radiomics features in the full dataset using the LASSO Cox regression model with ten-fold cross-validation (24). We obtained seven radiomics features (three signal intensity features and four texture features) that were most closely related to the prognosis. A radiomics signature was constructed based on the linear combination of the screened seven radiomics features and their corresponding Cox regression coefficient products. The radiomics signature we constructed is described by a formula in the Supplementary Material.

### 3.3 Correlation between radiomics signature and clinical information

The correlation between the radiomics signature and clinical information was evaluated using heat map analysis (Supplementary Figure S1). The results show that “GLCM_Contrast_Variance” has a high correlation with survival status, mostly in red color.

### 3.4 CoxPH model

**Figure 3:**
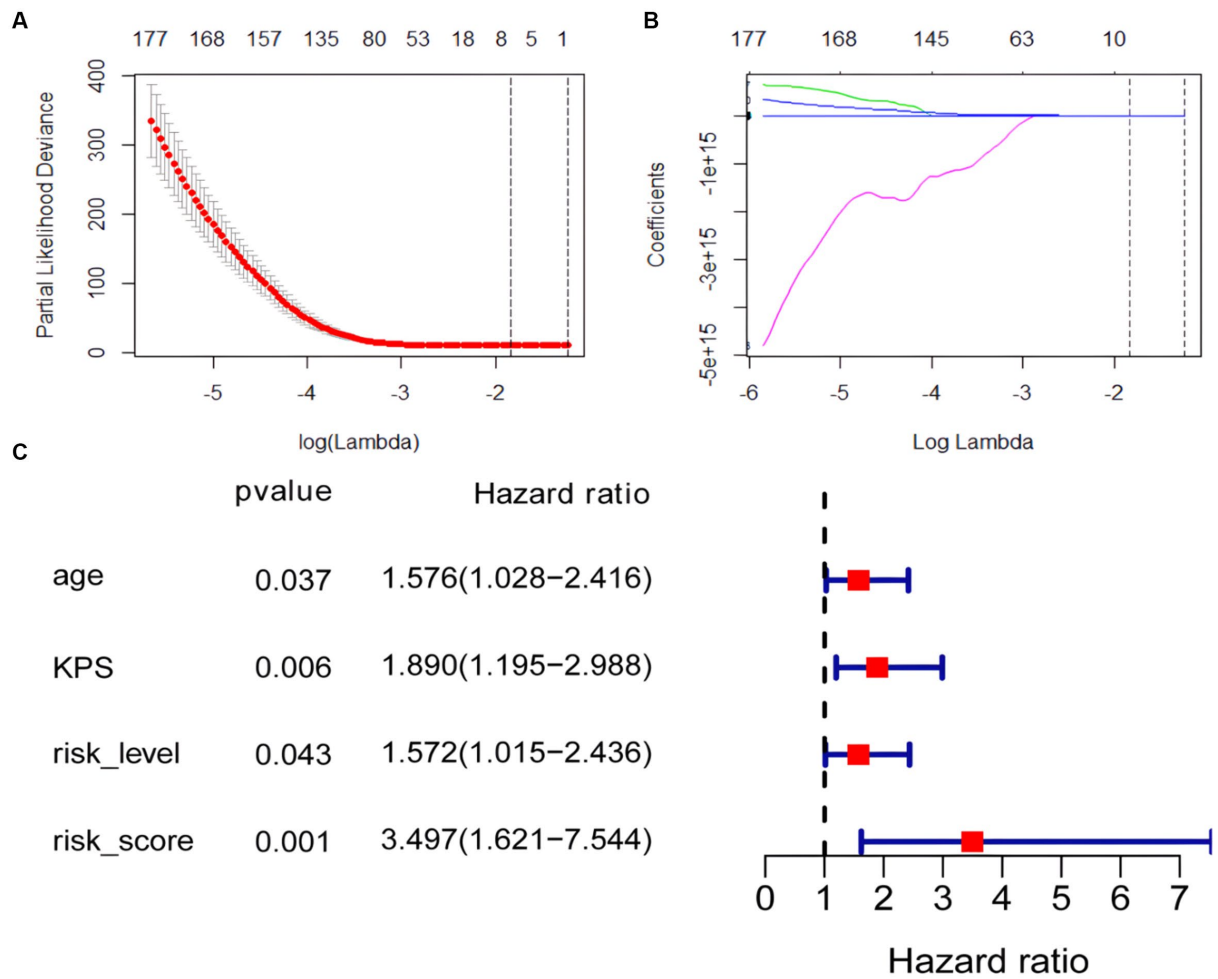
Coefficient convergence of LASSO Cox model for screening radiomics features and forest plot of univariate cox analysis. **(A)** The LASSO Cox model used tenfold cross-validation to select the optimal parameters. **(B)** The convergence of the coefficients of radiomics features under the parameters corresponding to the left figure, with each curve in the panel representing the trajectory of a feature coefficient. **(C)** Forest plot of univariate cox analysis.

**Figure 4:**
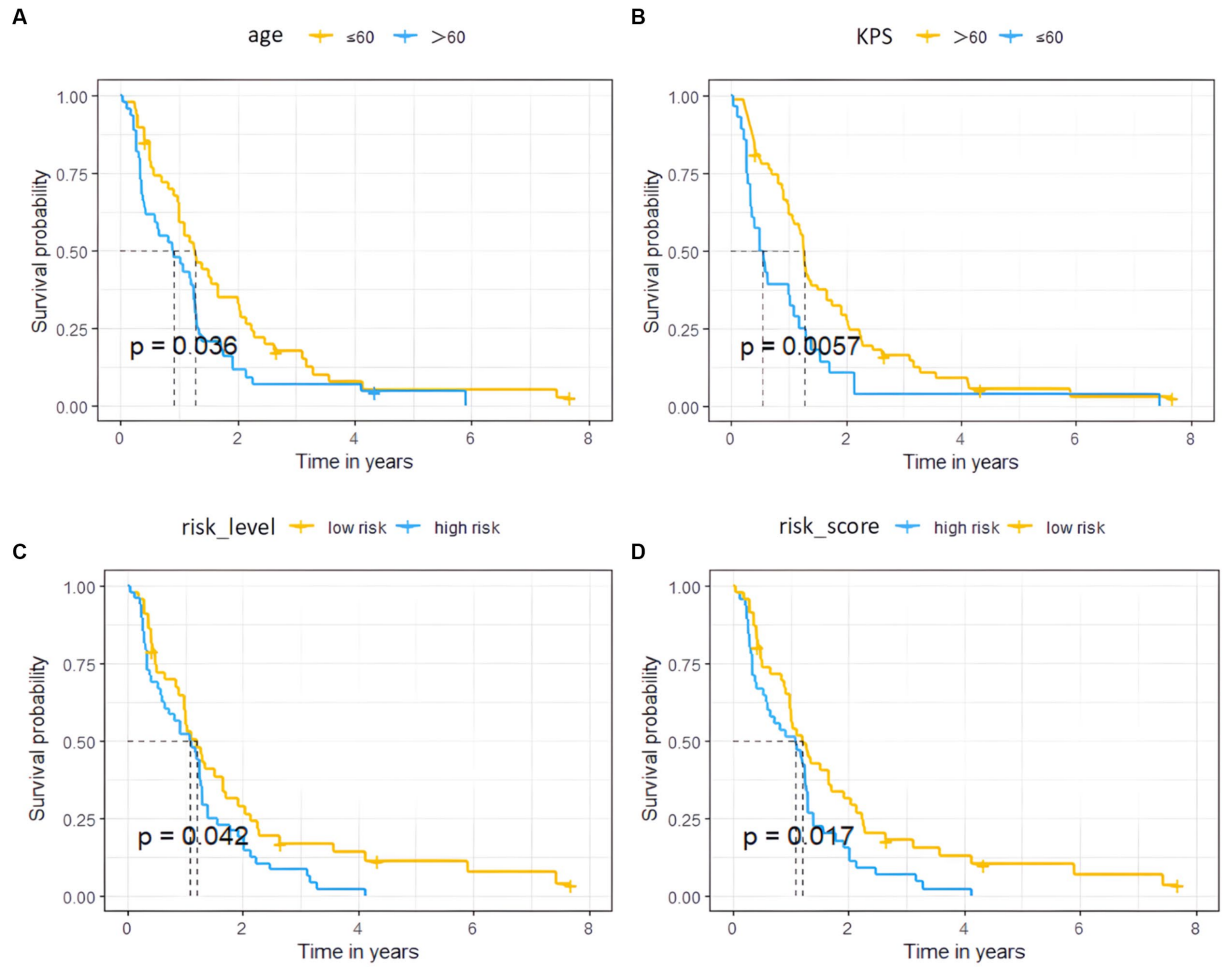
Survival curves of the high and low risk groups by univariate Cox analysis. **(A–D)** represent age, KPS, radiomics risk level, and radiomics risk score, respectively.

The univariate cox analysis showed that age (HR = 1.576, *p* = 0.037), KPS score (HR = 1.890, *p* = 0.006), radiomics risk score (HR = 3.497, *p* = 0.001), and radiomics risk level (HR = 1.572, *p* = 0.043) were prognostic factors for overall survival in GBM (Table 2), and the univariate analysis forest plot is shown in Figure 3; multivariate cox analysis showed that KPS score (HR = 1.864, *p* = 0.008), radiomics risk score (HR = 3.370, *p* = 0.003) were prognostic factors for overall survival of GBM (Table 2). In the training and test set, the C-index of the CoxPH model was divided into 0.663 and 0.635, with an overall C-index of 0.662, and for predicting 1-year, 3-year, and 5-year survival, the brier score was 0.225, 0.080, and 0.040, respectively, and the IBS was 0.102 (Table 3). The KM survival curves for variables that were significant for the univariate survival analysis are shown in Figure 4.

**Table 2:** Univariate and multivariate cox analysis of overall survival of GBM patients.

Variables	Univariate analysis		Multivariate analysis	
	Hazard ratio (95% CI)	*p* value	Hazard ratio (95% CI)	*p* value
Age	1.576 (1.028–2.416)	0.037	1.452 (0.943–2.235)	0.090
KPS	1.890 (1.195–2.988)	0.006	1.864 (1.175–2.956)	0.008
Risk level	1.572 (1.015–2.436)	0.043	1.041 (0.580–1.850)	0.090
Risk score	3.497 (1.621–7.544)	0.001	3.370 (1.499–7.573)	0.003

**Table 3:** Hyperparameters, C-index and IBS results for the five models.

Model	C-index		Hyperparameters	C-index	Brier score			IBS
	Training set	Test set			1-year	3-year	5-year	
CoxPH	0.663	0.635	none	0.662	0.225	0.080	0.040	0.102
Survival Tree	0.702	0.655	max_depth:5,min_samples_leaf:2,min_samples_split:12,n_estimators = 10	0.564	0.263	0.190	0.133	0.192
RSF	0.735	0.667	max_features:sqrt,min_samples_leaf = 2,min_samples_split = 4,n_estimators = 10	0.642	0.214	0.143	0.124	0.152
DeepSurv	0.882	0.732	num_nodes = [32,32],out_features = 1,dropout = 0.2,learning rate = 0.005	0.691	0.203	0.139	0.124	0.116
DeepHit	0.608	0.560	num_nodes = [32,32],out_features = labtrans.out_features,dropout = 0.1,learning rate = 0.001	0.617	0.348	0.330	0.146	0.261

### 3.5 SurvivalTree and RSF model

**Figure 5:**
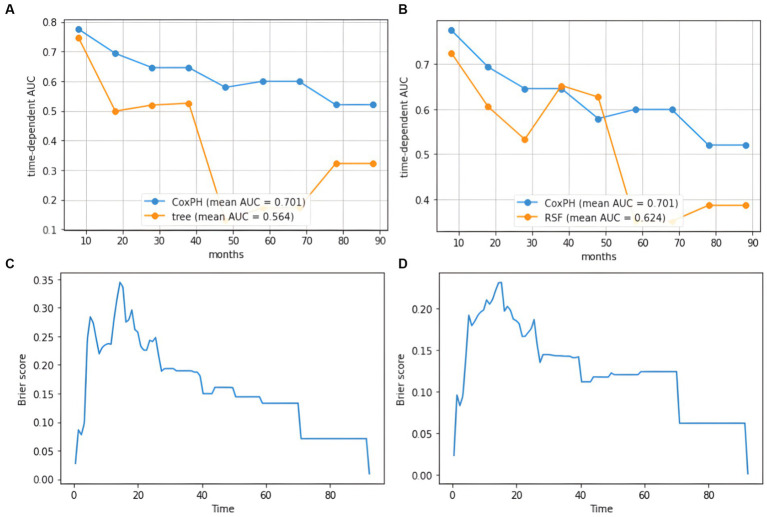
AUC plot of cumulative survival time and IBS diagram based on the tree model. **(A)** AUC results for the SurvivalTree model, **(B)** AUC results for the RSF model, **(C)** IBS results for the SurvivalTree model, **(D)** IBS results for the RSF model.

GBM survival prediction models based on the SurvivalTree and RSF tree algorithms were built using the training set and validated in the test set. Figure 5 shows the AUC values of the CoxPH model, the SurvivalTree model and the RSF model over time. As can be seen from the graph, the CoxPH model has the highest AUC value of 0.701, and the SurvivalTree model has the lowest AUC of 0.564.

In the training and test set, the C-index of the SurvivalTree model was divided into 0.702 and 0.655, and the overall C-index was 0.564. For predicting 1-year, 3-year, and 5-year survival, the brier scores were 0.225, 0.080, and 0.040, respectively, and the combined brier score was 0.192. In the training and test set, the C-index of the RSF model was divided into 0.735 and 0.667, and the overall C-index was 0.642; for predicting 1-year, 3-year, and 5-year survival, the brier scores were 0.214, 0.143, and 0.124, respectively, and the IBS was 0.152 (Table 3). The IBS plots of the two models are shown in Figure 5.

**Figure 6:**
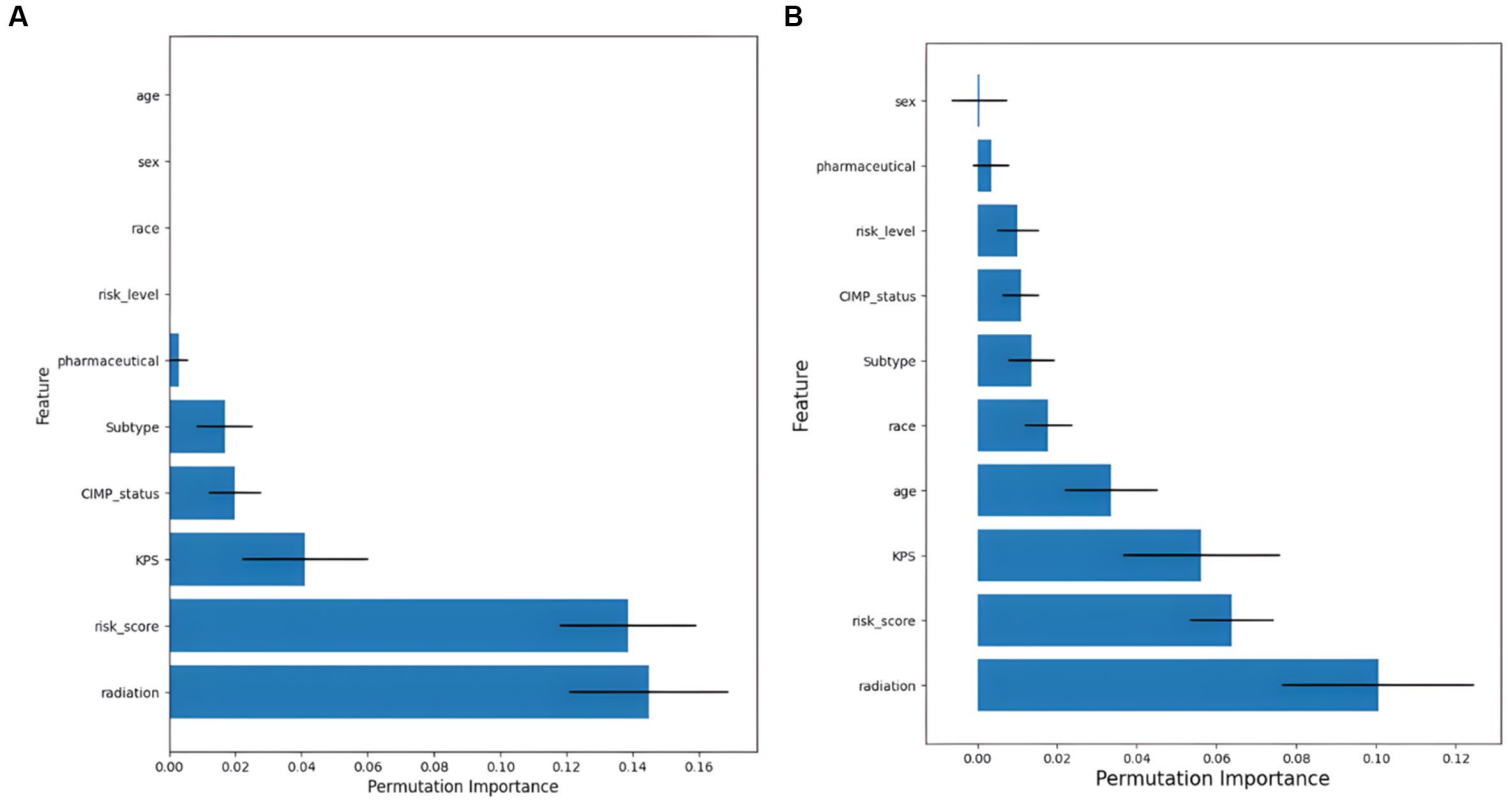
Feature importance ranking results. **(A)** Results of feature importance ranking for the SurvivalTree model, and **(B)** results of feature importance ranking for the RSF model.

The ranked importance of SurvivalTree and RSF model features are shown in Figure 6 and Supplementary Table S2; from the table, we can see that KPS, radiation and risk score are more important for the model. For both models, radiation is the most important feature, if radiation is removed from the model, the C-index of both will decrease by 0.145 and 0.101, respectively.

### 3.6 Deep learning model

**Figure 7:**
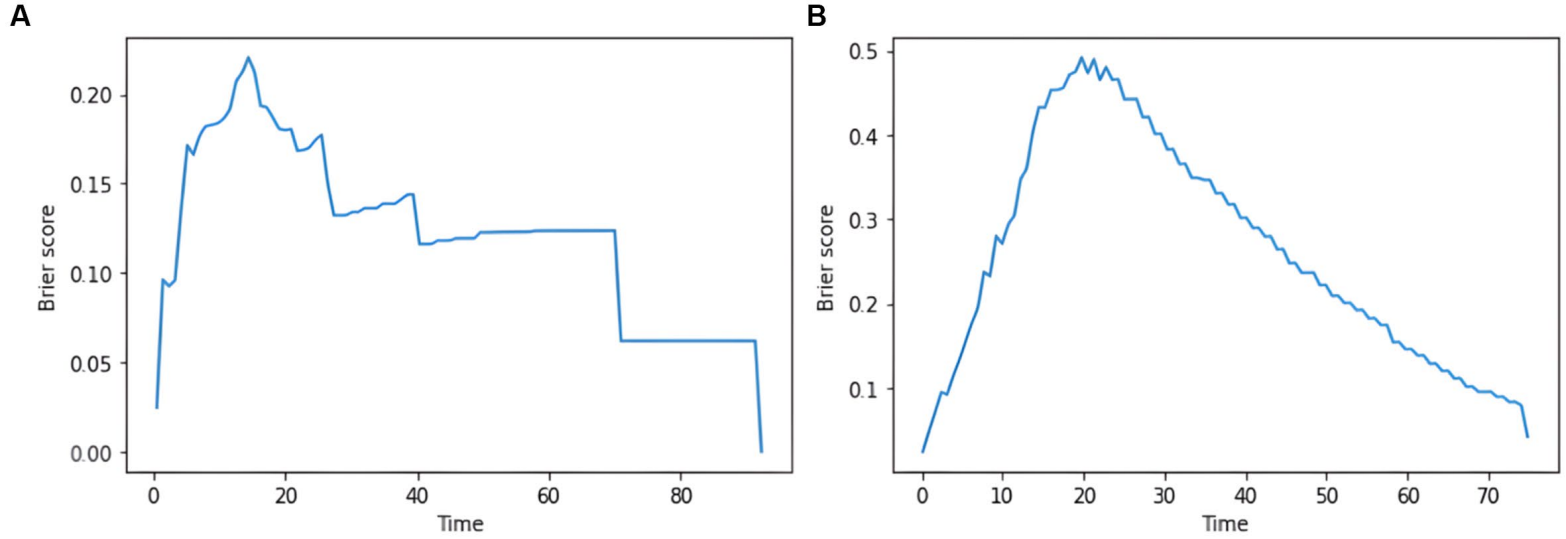
plot of the integrated brier score based on the deep learning model. **(A)** IBS results for the DeepSurv model. **(B)** IBS results for the DeepHit model.

DeepSurv and DeepHit survival prediction models based on deep learning algorithms were built using the training set and validated in the test set. In the training and test sets, the DeepSurv model had a C-index of 0.882 and 0.732, an overall C-index of 0.691, and a brier score of 0.203, 0.139, and 0.124 for predicting 1-year, 3-year, and 5-year survival, respectively, with a combined brier score of 0.116. In the training and test set, the DeepHit model had a C-index of 0.608 and 0.560, an overall C-index of 0.617, and a brier score of 0.347, 0.330, and 0.146 for predicting 1-year, 3-year, and 5-year survival, respectively, with an IBS of 0.261 (Table 3). The IBS plots for the two models are shown in Figure 7.

## 4 Discussion

Precision treatment of GBM can slow down tumour growth and help improve patient prognosis. Previous studies on GBM have used deep learning for diagnostic and prognostic assessment of tumours (17, 25). To our knowledge, this is the first study to use machine learning and radiomics approaches to assess the prognosis of GBM patients. In this study, by constructing radiomics prognostic labels, using different machine learning models and comparing the performance with the traditional CoxPH model, the results show that the DeepSurv deep learning model shows superior predictive power compared to the traditional CoxPH model.

While traditional radiography focuses on the visual presentation of images, radiomics focuses on the relationship between image phenotypes and biological features and has been widely used in tumour diagnosis and prognosis evaluation (4). Studies have shown that FLAIR sequences are more advantageous in showing the extent of tumour borders and edema and that 90% of GBM recurrence occurs in the peritumoral edema area and has been shown to correlate with the prognosis of GBM (26). The FLAIR sequence was superior in showing the extent of the tumour border and edema. Some progressive patients showed no significant enhancement on the contrast scan but showed a high signal on the FLAIR sequence (27). Therefore, it is important to explore the prognostic evaluation of non-contrast FLAIR sequences in GBM. In order to construct a radiomics prognostic signature, we used the LASSO cox regression model to reduce 1792 features to 7 potential predictive features. The results of this study showed that the seven radiomics features obtained in the FLAIR sequence were strongly associated with GBM survival, and these features indicated grey-scale heterogeneity of GBM. In addition, the radiomics risk score was shown to be an independent prognostic factor for GBM by cox univariate and multivariate analyses. The radiomics risk score was likewise the more important feature in the tree model-based feature importance ranking, suggesting that our constructed radiomics risk score can be used as a prognostic marker for GBM.

The CoxPH model is a classic approach to survival analysis and event prediction; however, the model is semi-parametric and assumes that the risk of an event is linearly related to the variables. Recently, tree-based models have received increasing attention from researchers in addressing the identification of multidimensional interactions. SurvivalTree is similar to decision trees because it is constructed by the recursive splitting of tree nodes. Compared to CoxPH, SurvivalTree is more relaxed in its requirements for survival information and does not require survival times to satisfy a specific distribution (21). RSF is a combination of random forest and SurvivalTree. The advantage of the RSF model is that it is not constrained by the assumptions of proportional risk and log-linearity (22). Also, it can prevent the overfitting problem of its algorithm through two random sampling processes (28). In our study, the SurvivalTree and the RSF model achieved a C-index of 0.70 or higher in the training set. However, as the survival tree model has fewer parameters available for adjustment and is not an integrated algorithm, it has a lower overall C-index. The IBS results for both models also showed that the RSF performed better. In addition, the AUC values for the cumulative survival times of the two models indicate a significant difference between the first and second half of the time horizon, with higher AUC values for the model in the first half of the time horizon and lower AUC values in the second half of the time horizon. Therefore, the models are most effective in predicting mid-term mortality.

Deep learning models can learn and infer higher-order nonlinear combinations between patient clinical outcomes and predictor variables in an entirely data-driven manner and have been shown to outperform standard survival analysis, with one advantage being the ability to discern complex relationships between clinical outcomes and predictor variables without prior feature selection (14). In this study, the DeepSurv model achieved the highest C-index in both the training and test set. At the same time, the overall C-index also indicated that the model was superior, suggesting that the deep learning-based survival model outperformed the CoxPH and RSF models in predicting GBM survival. Previous deep learning prognostic models based on clinical risk factors achieved a C-index of 0.823 and 0.700 in the training and test set, respectively (17); the present study achieved 0.882 and 0.667 in the training and test set, indicating the superior performance of the prognostic model based on radiomics features. Another deep learning model constructed in this study is DeepHit, which can directly learn the distribution of first death times and performs better in dealing with multiple competing risks (28). However, since the ending of this study is a dichotomous variable and there are no multiple competing risks, the performance of this model was not improved by hyperparameter tuning, and this model may not apply to our data structure.

There are limitations to this study. First, MRI images were collected retrospectively from the TCIA database, and the heterogeneity of different imaging parameters generated by different devices and field strengths could not be controlled. In addition, there was a relatively low number of patients in this study. Some patients also had incomplete clinical risk factors. Second, a large amount of redundant information in the sequence images leads to a considerable workload and subjectivity in manual segmentation. A more advanced approach is to use deep learning models such as CNN to learn features directly from images, which reduces the presence of subjectivity between the raters. Finally, to construct prognostic models, our study only extracted features from FLAIR images. In constructing the models, it did not make use of structural images or functional MRI techniques.

## 5 Conclusion

In conclusion, based on the TCGA and TCIA databases combined with a radiomics approach, this study confirmed that the DeepSurv model based on deep learning achieves better performance in GBM patient data compared to the CoxPH model. Based on the above-optimized model, a personalized treatment recommendation system for GBM can be developed to predict patient prognosis accurately.

## Data availability statement

The original contributions presented in the study are included in the article/Supplementary materials, further inquiries can be directed to the corresponding authors.

## Ethics statement

The study is based on the data available in the public domain to use; therefore, no ethics statement is required for this work.

## Author contributions

DZ: Data curation, Methodology, Software, Writing – original draft, Writing – review & editing. JL: Methodology, Software, Validation, Writing – original draft, Writing – review & editing. BL: Investigation, Software, Validation, Visualization, Writing – original draft. AY: Data curation, Project administration, Writing – original draft. KL: Data curation, Validation, Writing – original draft. PH: Data curation, Formal analysis, Writing – review & editing. XH: Conceptualization, Data curation, Methodology, Writing – original draft. HY: Conceptualization, Formal analysis, Investigation, Writing – original draft. AS: Writing – review & editing. GM: Funding acquisition, Supervision, Writing – review & editing, Writing – original draft. CZ: Funding acquisition, Methodology, Supervision, Writing – review & editing, Writing – original draft.

## Footnotes

^1^ https://tcga-data.nci.nih.gov/

^2^ https://wiki.cancerimagingarchive.net/

^3^ https://www.itk-snap.org/
