# Supplementary material for: Comparison of MRI radiomics-based machine learning survival models in predicting prognosis of glioblastoma multiforme
Source: Front Med (Lausanne). 2023 Nov 30;10:1271687. doi: 10.3389/fmed.2023.1271687 (PMC10720716; doi:10.3389/fmed.2023.1271687)
Supplement: Supplementary file 1 [file Data_Sheet_1.docx]

Supplementary Material

# Supplementary formula

radiomics signature = -2.753057e-01*GLCM_Contrast_Variance+

-2.603719e+02*GLRLM_ShortRunLowGreyLevelEmphasis_Offset_1_Min+

0.000e+00*GLRLM_ShortRunLowGreyLevelEmphasis_Offset_24_Min+

-9.867137e+00*GLSZM_ZoneSizeNoneUniformityNormalized_Min+

-2.198042e-03*Histogram_Bin-2_Frequency_Variance+

6.595832e-01*Intensity_MeanAbsoluteDeviation_Skewness+

-1.700838e+13*Morphologic_Eccentricity_Variance

# Supplementary Tables and Figures

## Supplementary Tables

**Supplementary Table 1.** List of predictor variables available for model training, number of missing instances and missing value handling techniques used.

| Prognostic  variable | Type of  variable | Number of missing instances | Handling technique employed |
| --- | --- | --- | --- |
| Age | Categorical | 0 | NA |
| Sex | Categorical | 0 | NA |
| Race | Categorical | 3 | KNN |
| KPS | Categorical | 0 | NA |
| Subtype | Categorical | 0 | NA |
| CIMP_status | Categorical | 8 | KNN |
| Radiation | Categorical | 0 | NA |
| Pharmaceutical | Categorical | 0 | NA |

**Supplementary Table 2**. Results of feature importance ranking for SurvivalTree and RSF models.

| Feature | SurvivalTree_weight | SurvivalTree_std | RSF_weight | RSF_std |
| --- | --- | --- | --- | --- |
| radiation | 0.145 | 0.024 | 0.101 | 0.024 |
| risk_score | 0.139 | 0.021 | 0.064 | 0.010 |
| KPS | 0.041 | 0.019 | 0.056 | 0.020 |
| age | 0.000 | 0.000 | 0.034 | 0.012 |
| race | 0.000 | 0.000 | 0.018 | 0.006 |
| Subtype | 0.017 | 0.008 | 0.014 | 0.006 |
| CIMP_status | 0.020 | 0.008 | 0.011 | 0.005 |
| risk_level | 0.000 | 0.000 | 0.010 | 0.005 |
| pharmaceutical | 0.003 | 0.003 | 0.004 | 0.005 |
| sex | 0.000 | 0.000 | 0.001 | 0.007 |

## Supplementary Figures

## **
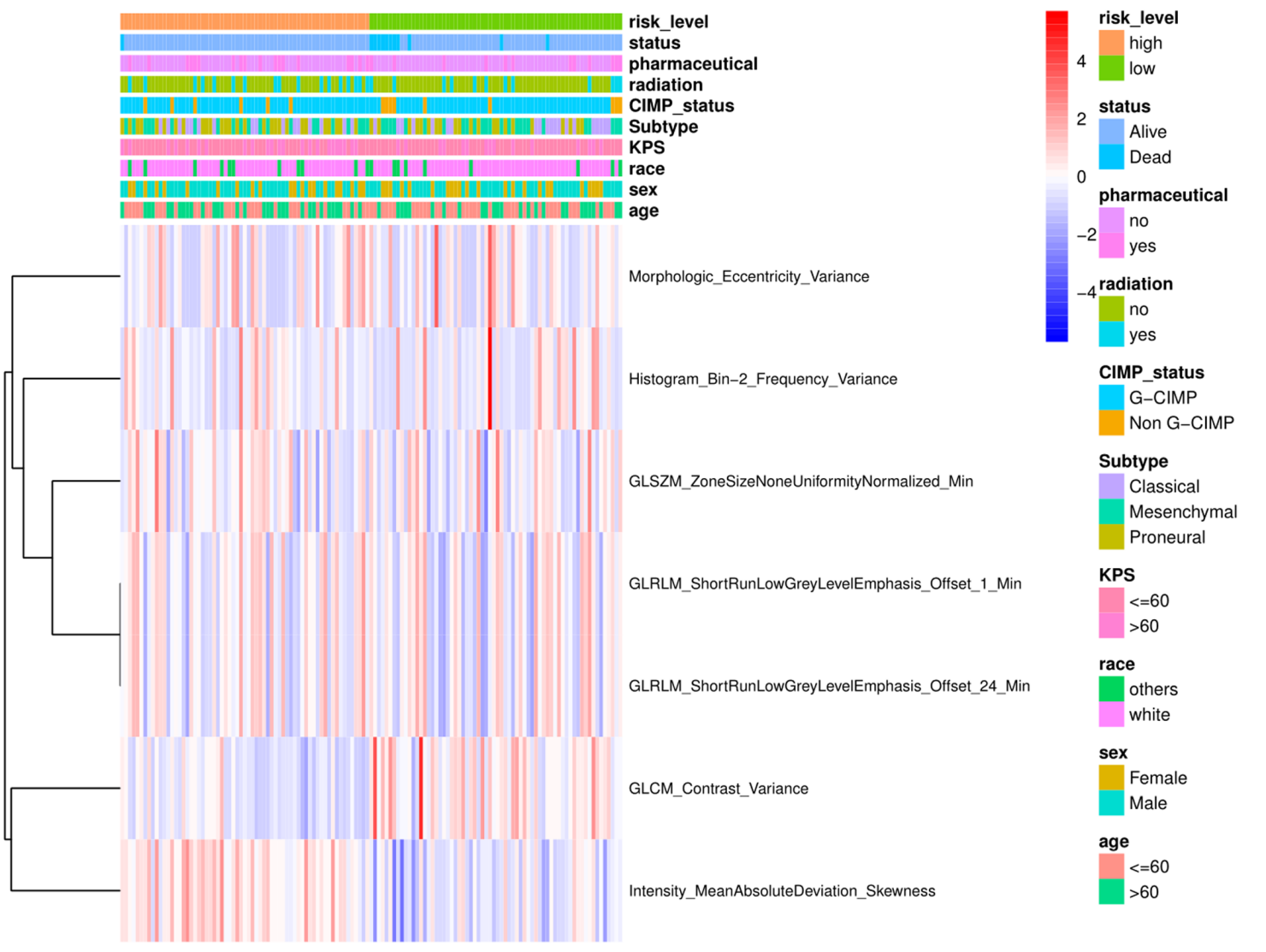
Supplementary Figure 1.** Heat map of clinical information and seven radiomics features. The x-axis is the effect of unsupervised cluster analysis of clinical information for GBM patients; the y-axis is the value of radiomics features.
